# Hypothermic Machine Perfusion vs. Static Cold Storage in Kidney Transplantation: A Retrospective Paired-Kidney Study from Latvia

**DOI:** 10.3390/medicina61091641

**Published:** 2025-09-10

**Authors:** Pāvils Plūme, Igors Losevs, Elizabete Anna Loseva, Aleksandrs Maļcevs, Vadims Suhorukovs, Olga Jegorova, Viktors Ševeļovs, Jānis Jušinskis

**Affiliations:** 1 Latvian Transplant Center, Pauls Stradiņš Clinical University Hospital, LV-1002 Riga, Latvia; 2 Department of Surgery, Riga Stradiņš University, LV-1007 Riga, Latvia; 3 Faculty of Medicine and Life Sciences, University of Latvia, LV-1586 Riga, Latvia

**Keywords:** kidney transplant, hypothermic machine perfusion, delayed graft function, nephrology, urology

## Abstract

*Background and Objectives*: Kidney transplantation remains the gold-standard treatment for end-stage renal disease (ESRD). For deceased donor transplantation, optimal allograft preservation represents a critical determinant of success. While static cold storage (SCS) has been the historical standard, hypothermic machine perfusion (HMP) has emerged in recent decades as a technologically advanced alternative. However, comparative data from smaller-volume centers utilizing exclusively donation after brain death (DBD) donors remain scarce. Materials and Methods: This retrospective single-center study included 94 patients who received kidney transplants from deceased DBD donors between January 2018 and December 2024. We employed a paired kidney study design where one kidney from each donor was preserved using HMP (LifePort, set at 30/20 mm Hg pressure), while the contralateral kidney was stored in SCS. Parameters compared were creatinine concentration in recipient serum after transplantation, DGF, acute rejection and hospital stay. *Results*: The HMP group had a significantly longer cold ischemia time (CIT) (18.09 ± 5.91 h, range: 6.5–34.0 h) compared to the SCS group (12.36 ± 5.18 h, range: 4.0–23.0 h; *p* < 0.005). The DGF rate was significantly lower in the HMP group (4.3%) than the SCS group (25.5%) (*p* = 0.004). HMP was also associated with a shorter mean hospitalization (11.81 vs. 15.66 days, *p* = 0.008) and superior early graft function, particularly in kidneys with CIT ≥ 18 h, which showed significantly lower serum creatinine at day 14 (124.48 vs. 164.89 µmol/L, *p* = 0.036). *Conclusions*: HMP usage in kidney transplantation decreased the possibility for DGF in DBD donors and shortened the post-op hospitalization time. It is a feasible method for kidney storage before transplantation even in a case of prolonged CIT.

## 1. Introduction

Kidney transplantation remains the gold-standard treatment for end-stage renal disease (ESRD), demonstrating superior patient survival and outcomes compared to long-term dialysis [1,2]. Transplant organs are sourced from either living donors—which is associated with favorable outcomes—or deceased donors [3]. Deceased donation occurs after either circulatory death (DCD) or brain death (DBD); this article focuses exclusively on the latter.

The preservation of these allografts between procurement and implantation is a critical determinant of transplant success. While static cold storage (SCS) has been the historical standard, hypothermic machine perfusion (HMP) has emerged in recent decades as a technologically advanced alternative, with trials demonstrating significant reductions in delayed graft function (DGF) rates and improvements in long-term graft survival [4,5].

The protective mechanisms of HMP are multifactorial. By maintaining continuous flow, HMP exerts beneficial shear stress on the vascular endothelium, promoting vasodilation via nitric oxide release and improving microcirculation. Furthermore, HMP reduces cellular metabolism and mitigates ischemia–reperfusion injury by flushing out metabolic waste. It also appears to modulate the immune response by reducing pro-inflammatory cytokine levels [6,7,8]. Despite this evidence, comparative data on HMP’s efficacy from smaller-volume centers that utilize exclusively DBD donors remain scarce.

At Pauls Stradiņš Clinical University Hospital (Riga, Latvia), our transplant program performs approximately 60–70 kidney transplantations annually, with 95% originating from DBD donors, with brain death confirmed by a 24 h protocol and consent provided via a soft opt-out system. Latvia’s unique geographic challenges—including frequent donor procedures at regional hospitals resulting in prolonged organ transport times (>3 h) and demographic factors necessitating broader use of extended criteria donors (ECD)—have prompted our center to implement a selective use of LifePort HMP technology. From 2018 to 2024, we employed HMP for 47 kidney allografts. This retrospective study compares early postoperative outcomes—including the incidence of DGF and serum creatinine levels—as well as 12-month graft function between HMP—preserved and SCS-preserved kidney allografts from DBD donors within our institutional cohort.

## 2. Materials and Methods

This retrospective, single-center study included 94 patients who underwent kidney transplantation from DBD donors at the Latvian Transplantation Center, Pauls Stradiņš Clinical University Hospital, between January 2018 and December 2024.

Due to institutional availability of only one LifePort machine, we employed a paired kidney study design where one kidney from each donor was preserved using HMP (Organ recovery systems Country, Itasca, IL, USA, Supplier city, Diegem, Belgium) (LifePort, set at 30/20 mmHg pressure), while the contralateral kidney was stored in SCS. This approach allowed direct comparison of preservation methods as both kidneys came from the same donor. SCS utilized either StoreProtect Plus or Custodiol HTK as the preservation solution while for HMP we used Pumpprotect.

Donors were classified as ECD if they were >60 years old or >50 years old with at least two of the following: (1) history of hypertension, (2) serum creatinine ≥1.5 mg/dL, or (3) death from cerebrovascular accident [4]. Standard criteria donors (SCD) comprised all other cases.

The immunosuppressive protocol for all kidney transplant recipients consisted of induction therapy with methylprednisolone plus either basiliximab or anti-thymocyte globulin (ATG), followed by a maintenance triple-drug regimen of cyclosporine, mycophenolate mofetil, and prednisolone. For anticoagulation, patients received an initial unfractionated heparin infusion (400 IU/h) for the first 48 h postoperatively, which was then transitioned to a standard regimen of low-molecular-weight heparin (LMWH) for two weeks.

Exclusion criteria were recipients and donors younger than 18 years, cases not paired, early vascular complications such as artery thrombosis, dissection or bleeding mandating reoperation and primary non function (2 cases from one donor), cases of non-function of the Life Port machine—resulting in switch to a SCS (defect in cassette of Life port device was discovered).

Primary outcomes included DGF, defined as requiring dialysis within the first postoperative week, and acute rejection episodes diagnosed through clinical, biochemical, and histological assessment during follow-up.

The statistical analysis was performed using IBM SPSS Statistics (version 27.0). The Shapiro–Wilk test was used to assess the normality of continuous variables, and Levene’s test was used to evaluate the homogeneity of variances. As the assumptions of normality and homogeneity of variances were met (*p* > 0.05 for both), parametric methods were employed for subsequent analysis.

To minimize selection bias and ensure comparability between the groups, a 1:1 propensity score matching (PSM) analysis was performed. The matching was based on the following covariates: donor age, recipient age, donor gender, and recipient gender.

The comparison of outcomes was conducted using the following tests: McNemar’s test was used for paired categorical data (e.g., comparing the incidence of Delayed Graft Function (DGF) between the matched HMP and SCS pairs, and for assessing the balance of donor and recipient gender after matching). The Chi-square (χ^2^) test was used for independent (unpaired) categorical data when comparing subpopulations (e.g., comparing the frequency of re-transplantation in the original, unmatched cohorts). Results are presented as odds ratios (OR) and relative risk (RR) with corresponding 95% confidence intervals. Paired-samples *t*-test was used for continuous variables.

To further adjust for potential confounders and analyze the influence of multiple factors on the outcomes, multivariable linear regression (for continuous outcomes) and logistic regression (for binary outcomes) models were constructed. All regression models were checked for multicollinearity using the variance inflation factor (VIF); a VIF value < 5 was considered to indicate no significant multicollinearity.

## 3. Results

### 3.1. Donor Population

A total of 94 cases were included in the study. Kidneys were always obtained from the same donor—one preserved via HMP and the other in SCS, resulting in 47 pairs. The mean donor age was 52 years (30–74; SD—9.9072) with a mean intensive care unit (ICU) stay of 3 days (1–14; SD—2.2376). At the time of organ procurement, the mean serum creatinine concentration was 106.19 µmol/L (35–408; SD—63.3), and the mean sodium concentration was 156.8 mmol/L (138–178; SD—9.253). Among the donors, 56 met the standard criteria, while 38 were classified as ECD. Detailed donor characteristics are summarized in Table 1.

### 3.2. Recipient Population

The study included 37 female and 57 male recipients, with a mean age of 50 years. The most common indication for transplantation was end-stage renal failure due to glomerulonephritis, IgA nephropathy, and autosomal dominant polycystic kidney disease (ADPKD). Primary graft function (PGF) was observed in 80 recipients, while DGF occurred in 14 cases. For 82 recipients, this was their first kidney transplant; 10 had undergone a second transplant, and 2 had received a third transplant. The mean skin-to-skin transplant duration was 171.15 min, and the mean hospitalization time was 14.35 days. Detailed recipient characteristics are summarized in Table 2.

### 3.3. Comparing Impact of CIT on Serum Creatinine

**Figure 1:**
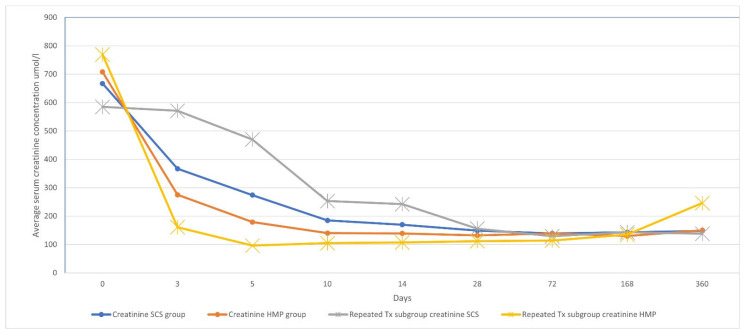
Serum creatinine concentration changes in dynamics.

All patients were evaluated for serum creatinine changes up to 14 days postoperatively (see Table 3 and Figure 1). After applying a Bonferroni correction for the four primary time points analyzed (days 5, 10, 14 and 12 months), a significantly lower serum creatinine level was observed in the HMP group only on postoperative day 5 (179.06 µmol/L vs. 274.94 µmol/L, *p* = 0.044). No significant differences were found between the groups at the other time points.

The effect of cold ischemia time (CIT) on serum creatinine levels was analyzed by stratifying data into two cohorts: CIT < 18 h and CIT ≥ 18 h. In recipients with CIT < 18 h, we found no statistically significant difference in serum creatinine between the HMP and SCS groups at any time point. In recipients with CIT ≥ 18 h, mean serum creatinine was significantly lower in the HMP group at day 14 (124.48 vs. 164.89 µmol/L; *p* = 0.036). However, we found no statistically significant differences on day 5, 10 or 12 months after the transplantation.

### 3.4. Comparing Impact of CIT on DGF

The overall mean cold ischemia time (CIT) was 15.23 ± 6.23 h (range: 4.0–34.0 h). The HMP group exhibited a significantly longer CIT (18.09 ± 5.91 h, range: 6.5–34.0 h) compared to the SCS group (12.36 ± 5.18 h, range: 4.0–23.0 h; *p* < 0.005). When assessing the impact of CIT on DGF using an 18 h threshold, no statistically significant difference was found in its rate within either the HMP (*p* = 0.325) or SCS (*p* = 0.259) groups.

### 3.5. Differences in Post Op Hospitalization Time

When evaluating hospitalization duration, it was significantly shorter in the HMP group (11.81 days) compared to the SCS group (15.66 days; *p* = 0.008). See Table 4.

### 3.6. Effect on DGF

**Figure 2:**
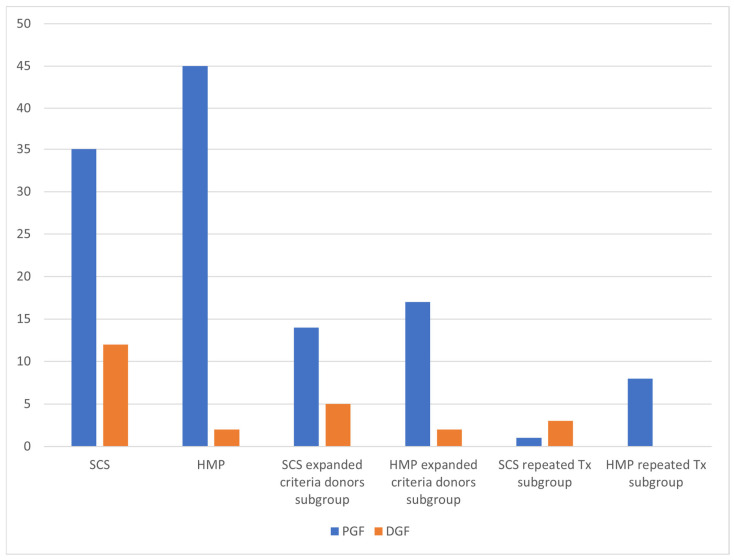
DGF frequency.

DGF occurred in 2 cases of HMP group versus 12 cases of the SCS group (*p* = 0.004), with an odds ratio of 0.130 (95% CI: 0.027–0.617). This protective effect of HMP was also significant in the subgroup of recipients undergoing repeat transplantation (*p* = 0.018). See Table 5 and Figure 2.

### 3.7. Subgroup Analysis

Subgroup analyses revealed no significant differences in DGF or serum creatinine trends between SCDs and ECDs. Acute rejection episodes occurred in 4 cases in the HMP group versus 7 cases in the SCS group (*p* = 0.261). Donor ICU stay exceeding 3 days was observed in 26 cases (13 per group) but had no impact on graft function, acute rejection rates, or hospitalization duration.

### 3.8. Potential Confounders

A multivariable analysis was performed to identify risk factors for delayed graft function (DGF). Among the parameters evaluated, only hypothermic machine perfusion (HMP) demonstrated a statistically significant association (*p* = 0.006; see Table 6). When acute rejection was used as the primary outcome, a history of previous transplantation was the only parameter that reached statistical significance (OR [95% CI], *p* = 0.031; tolerance = 0.916, VIF = 1.092). In a model evaluating predictors of prolonged hospitalization length of stay, two factors were significantly associated: HMP (*p* < 0.001; tolerance = 0.759, VIF = 1.318) and cold ischemia time (CIT) (*p* = 0.031; tolerance = 0.673, VIF = 1.486).

### 3.9. Propensity Score Matching

Propensity score matching was based on following covariates: donor age, donor gender, recipient age, recipient gender, number of kidney transplants (Re-transplantation), DGF.

In all pairs the distribution was not statistically significant. See Table 7 and Table 8.

After propensity score matching, our analysis confirmed a statistically significant difference in the primary outcome, the incidence of Delayed Graft Function (DGF), which was significantly lower in the HMP group compared to the SCS group (4.3% vs. 25.5%, *p* = 0.013).

## 4. Discussion

### 4.1. Design

Our study used a paired-kidney design, in which both kidneys from the same donor were allocated to different preservation methods—one to HMP and the contralateral kidney to SCS. This approach minimized variability among donors, allowing for a direct comparison of preservation techniques. A similar methodology was employed by Moers et al. (*NEJM*, 2009) [4] and Pablo Domínguez López et al. (Experimental and Clinical Transplantation, 2024) in studies involving donation after cardiac death (DCD) kidneys [9]. In contrast, our study exclusively enrolled DBD donors and did not randomize preservation assignments.

### 4.2. Key Findings

Our study demonstrates that HMP significantly reduced DGF rates (2 vs. 12 cases; *p* = 0.004) and shortened hospitalization duration (11.81 vs. 15.66 days; *p* = 0.008) compared to SCS. Notably, HMP mitigated the detrimental effects of prolonged CIT on early graft function, as evidenced by lower serum creatinine levels on postoperative day 5 (179.06 µmol/L vs. 274.94 µmol/L, *p* = 0.044). The significant reduction in serum creatinine in the HMP group on day 14 was exclusively observed in the cohort with CIT ≥ 18 h. This suggests that the benefit of HMP—likely through the reduction in endothelial damage, reducing the expression of key pro-inflammatory cytokines and improvement of microvascular flow—becomes most clinically relevant when grafts are exposed to prolonged ischemic stress [6]. The lack of a significant difference in the shorter CIT cohort implies that for kidneys with already brief preservation times, the additional benefit of HMP over SCS may be marginal. These findings align with multiple trials [10,11,12], supporting HMP’s role in optimizing outcomes, particularly in settings with logistical challenges such as prolonged organ transport. Unlike prior studies that assessed serum creatinine levels at discharge, we evaluated early postoperative creatinine trends, which correlated more closely with hospitalization duration (11.86 days for HMP vs. 15.66 days for SCS) [13]. These findings are similar to those reported by Sedigh et al. (Transplantation Proceedings, 2013) [14], who observed a statistically significant disparity in creatinine levels on day of discharge (120 vs. 130 µmol/L; *p* = 0.046) in their cohort of 139 recipients. In our study, no statistically significant differences were observed after post-operative day 5. However, by day 10, a trend toward significance emerged, with a creatinine disparity of 140.13 vs. 185.19 µmol/L (*p* = 0.088).

While our study confirmed a significant reduction in DGF with HMP (OR: 0.130; 95% CI: 0.027–0.617), subgroup analyses revealed no differential effect based on donor type (SCD vs. ECD) or CIT duration. This contrasts with literature emphasizing HMP’s advantages for ECDs [15], possibly due to our limited ECD subgroup (*n* = 38) or relatively short CIT in both groups (mean: 18.09 ± 5.91 h for HMP vs. 12.36 ± 5.18 h for SCS).

Notably, prolonged CIT did not increase the risk of DGF in kidneys preserved via HMP (*p* = 0.325), supporting its safety for extended storage. These findings align with those reported by Guy et al. (Experimental and Clinical Transplantation, 2015), who demonstrated comparable outcomes between kidneys preserved using HMP for 23 h and those preserved via SCS for 13 h [11]. However, the optimal CIT threshold for HMP remains undefined and warrants further investigation. Additionally, Gill et al. (Transplantation, 2013) observed a reduction in DGF with HMP across all preservation durations in SCD’s, as well as in extended criteria ECD’s with CIT exceeding 6 h and in DCD donors within the 6-to-24 h CIT range [16].

In our study, the longer CIT in HMP group was linked with the availability of operating theaters, usually performing both transplants in back-to-back fashion by the same team.

Our study found no statistically significant differences regarding acute rejection between the two groups. While the existing literature has predominantly examined HMP in the context of DGF and long-term graft survival, its influence on acute rejection remains uncertain. Intriguingly, however, Carlos Verdiales et al. (*World Journal of Transplantation*, 2024) observed that HMP was associated with a reduced incidence of acute rejection—a paradoxical effect that was particularly pronounced in cases with *prolonged* CIT. This finding suggests that HMP may mitigate ischemia-related injury more effectively in extended CIT scenarios, potentially through the downregulation of inflammatory mediators and vasoconstrictive peptides [17].

### 4.3. Limitations

This study has several important limitations. The single-center, retrospective design with a relatively small sample size (*n* = 47 pairs) may affect the generalizability of our findings to broader transplant populations. While the paired-kidney design helps control for donor-related variables, the non-randomized allocation of preservation methods could introduce potential selection bias. During the research, we had one life port machine available for each case. The allocation of kidneys to HMP was non-randomized and conducted at the operating surgeon’s discretion, based on factors such as anatomical suitability.

The absence of long-term follow-up data beyond 12 months precludes definitive conclusions regarding the impact of HMP on chronic allograft injury and long-term graft survival.

Additionally, the relatively short CIT in both groups (mean 18.09 h for HMP versus 12.36 h for SCS) may have limited our ability to detect more pronounced differences between preservation methods that might become apparent with prolonged ischemia times.

Another potential limitation is the exclusion of two transplant procedures due to HMP device malfunction, which may raise concerns of selection bias. It is critical to note that in both instances, the failure of the machine’s pumping mechanism meant the kidney was preserved solely by the surrounding ice-slush (0–4 °C) in the machine’s reservoir, effectively converting the preservation method to static cold storage (SCS). The objective of our study was to compare the efficacy of active HMP against SCS. Thus, the exclusion was necessary to ensure the integrity of the group comparisons.

Although the use of two different SCS solutions was a potential confounder, multivariable regression analysis revealed no significant association between solution type (StoreProtect Plus vs. Custodiol) and the incidence of DGF.

## 5. Conclusions

In this paired-kidney analysis, HMP was associated with a lower incidence of DGF and a reduced postoperative hospital length of stay compared to static cold storage (SCS). Multivariable analysis confirmed HMP as an independent, modifiable factor associated with a lower risk of DGF, underscoring its potential to improve outcomes irrespective of other donor or recipient variables. The implementation of hypothermic machine perfusion is an effective strategy to enhance early graft function. No differences were observed in outcomes between SCD and ECD or in cases with prolonged donor ICU stays. Further studies should investigate the benefits of HMP in prolonged CIT settings to establish optimal storage duration limits and refine clinical utilization protocols.

## Figures and Tables

**Table 1:** Donor population data.

	**Frequency**	**Percent**
Female	48	51.1%
Male	46	48.9%
Standard criteria donors	56	59.6%
Extended criteria donors	38	40.4%
Total	94	100.0%
	**Mean**	**Standard deviation**
Age (years) Weight (kg) Height (cm)	52.096 (30–74)	9.9072
	82.45 (45–135)	18.951
	173.04 (1620–190)	7.699
ICU stay (days) Serum creatinine concentration (µmol/L) Natrium concentration (mmol/L)	3.064 (1.0–14.0)	2.2376
	106.19 (35–408)	63.322
	156.38 (138–178)	9.253

**Table 2:** Recipient population data.

**Recipient Sex**	**Frequency**	**Percent**
Male	57	60.6%
Female	37	39.4%
Total	94	100%
**Recipient primary disease**		
Glomerulonephritis	17	18.1%
IgA nephropathy	15	16%
ADPKD	14	14.9%
Diabetic nephropathy Chronic interstitial nephritis	8 8	8.5% 8.5%
Reflux nephropathy	7	7.4%
Hypertensive nephropathy	6	6.4%
Glomerular sclerosis	5	5.3%
KSD	2	2.1%
Other	12	12.8%
**Recipient by graft function**		
Primary graft function	80	85.1%
Delayed graft function	14	14.9%
** Kidney transplant count **		
1 transplantation	82	87.2%
2 transplantations	10	10.6%
3 transplantations	2	2.1%
Total	94	100.0%

**Table 3:** Serum creatinine changes after transplant.

Paired Samples Statistics					Paired Samples Correlations		Paired Samples Test		
Serum Creatinine Concentration per Day (µmol/L)	Study Group	*n*	Mean	Std. Deviation	Correlation	*p*	Mean Difference	Std. Deviation	*p*
Day 5	HMP	47	179.06	138.82	0.264	0.073	−95.87	247.08	0.044
	SCS	47	274.94	244.23					
Day 10	HMP	47	140.13	73.075	0.283	0.054	−45.06	130.47	0.088
	SCS	47	185.19	130.75					
Day 14	HMP	47	138.74	62.80	0.165	0.268	−31.04	105.20	0.196
	SCS	47	169.79	95.39					
12 months	HMP	47	150.46	169.89	0.251	0.114	2.95	165.41	1
	SCS	47	147.51	60.27					

**Table 4:** Post-op hospitalization time.

Paired Samples Statistics					Paired Samples Correlations		Paired Samples Test		
**Post-surgery time (days)**	**Study Group**	***n***	**Mean**	**Std. Deviation**	**Correlation**	***p***	**Mean Difference**	**Std. Deviation**	***p***
	HMP	47	11.81	4.533	−0.24	0.871	−3.581	9.551	0.008
	SCS	47	15.66	8.297					

**Table 5:** DGF versus PGF in all subgroups.

	PGF	DGF	Total	Odds Ratio for DGF HMP vs. CS (95% Confidence Interval)	Relative Risk for DGF CS vs. HMP	*p* Value
**SCS (all)**	35 (74.5%)	12 (25.5%)	47 (100%)		6.000	0.004
**HMP (all)**	45 (95.7%)	2 (4.3%)	47 (100%)	0.130 (0.027–0.617)		
Total	80 (85.1%)	14 (14.9%)	94 (100%)			
**SCS ECD**	14 (73.7%)	5 (26.3%)	19 (100%)		2.500	0.202
**HMP ECD**	17 (89.5%)	2 (10.5%)	19 (100%)	0.329 (0.055–1.965)		
Total	31 (81.6%)	7 (18.4%)	38 (100%)			
**SCS (ICU stay > 3 days)**	11 (85.6%)	2 (15.4%)	13 (100%)		2.000	0.500
**HMP (ICU stay > 3 days)**	12 (92.3%)	1 (7.7%)	13 (100%)	0.458 (0.036–5.789)		
Total	23 (88.5%)	3 (11.5%)	26 (100%)			
**SCS (repeated transplant)**	1 (25%)	3 (75%)	4 (100%)			
**HMP (repeated transplant)**	8 (100%)	0 (0%)	8 (100%)			0.018
Total	9 (75%)	3 (25%)	12 (100%)			

**Table 6:** Multivariable analysis of risk factors for delayed graft function (DGF).

Linear Regression				Logistic Regression	
	*p*	Collinearity Statistics		Variables in the Equation	
**Constant**	0.940	**Tolerance**	**VIF**	***p***	**Exp(B)**
Donor age (years)	0.503	0.333	3.000	0.793	1.021
ECD	0.177	0.331	3.023	0.460	0.348
Donor gender	0.451	0.948	1.055	0.090	0.178
ICU stay > 3 days	0.649	0.827	1.209	0.436	2.427
HMP	0.006	0.743	1.346	0.012	31.927
CIT	0.836	0.667	1.498	0.547	1.059
Perfusion solution	0.108	0.850	1.177	0.073	0.113
Recipient gender	0.426	0.885	1.130	0.241	0.296
Kidney transplant count	0.079	0.907	1.102	0.126	6.113
Primary diagnosis	0.268	0.905	1.104	0.901 (for all diagnoses)	Not significant for all diagnoses

**Table 7:** Propensity score matching.

Paired Samples Statistics					Paired Samples Test		
Pair	Study Group	*n*	Mean	Std. Deviation	Mean	Std. Deviation	*p*
Donor age	HMP	47	52.096	9.9606	NA	NA	NA
	SCS	47	52.096	9.9606			
Recipient age	HMP	47	51.06	73.075	1.149	15.492	0.614
	SCS	47	49.91	130.753			

**Table 8:** Propensity score matching.

	**Donor Gender SCS**				***p* Value (McNemmer)**
Donor gender HMP		Female	Male	Total	1
	Female	24	0	24	
	Male	0	23	23	
	Total	24	23	47	
	**Recipient gender SCS**				*p* value (McNemmer)
Recipient gender HMP		Female	Male	Total	1
	Female	10	8	18	
	Male	9	20	29	
	Total	19	28	47	
	**Repeated Tx SCS**				*p* value (McNemmer)
Repeated transplantation HMP		NO	YES	Total	0.289
	NO	37	2	39	
	YES	6	2	8	
	Total	43	4	47	
	**DGF SCS**				*p* value (McNemmer)
DGF HMP		NO	YES	Total	0.013
	NO	33	12	45	
	YES	2	0	2	
	Total	35	12	47	

## Data Availability

The data used in this study are not publicly available due to ethical restrictions. However, they can be made available upon reasonable request to the corresponding author for specific research purposes, following all necessary agreements or approvals.

## Abbreviations

The following abbreviations are used in this manuscript:

ESRD	end stage renal disease
SCS	static cold storage
HMP	hypothermic machine perfusion
DGF	delayed graft function
DBD	donation after brain death
SCD	standard criteria donors
DCD	donation after cardiac death
ECD	extended criteria donors
CIT	cold ischemia time
ADPKD	autosomal dominant polycystic kidney disease
KSD	kidney stone disease
ICU	intensive care unit
OR	odds ratio
RR	relative risk

## References

[1] Shi B., Ying T., Chadban S.J. (2023). Survival after kidney transplantation compared with ongoing dialysis for people over 70 years of age: A matched-pair analysis. Am. J. Transplant..

[2] Strohmaier S., Wallisch C., Kammer M., Geroldinger A., Heinze G., Oberbauer R., Haller M.C. (2022). Survival benefit of first single-organ deceased donor kidney transplantation compared with long-term dialysis across ages in transplant-eligible patients with kidney failure. JAMA Netw. Open.

[3] Sharma V., Roy R., Piscoran O., Summers A., van Dellen D., Augustine T. (2020). Living donor kidney transplantation: Let’s talk about it. Clin. Med..

[4] Moers C., Smits J.M., Maathuis M.H., Treckmann J., van Gelder F., Napieralski B.P., van Kasterop-Kutz M., van der Heide J.J., Squifflet J.P., van Heurn E. (2009). Machine perfusion or cold storage in deceased-donor kidney transplantation. N. Engl. J. Med..

[5] Cojuc-Konigsberg G., Chopra B. (2025). Kidney machine perfusion is associated with improved long-term graft survival mediated by reduced delayed graft function: A mate-kidney analysis. Artif. Organs.

[6] Tatsis V., Dounousi E., Mitsis M. (2021). Hypothermic Machine Perfusion of Kidney Transplant: A Mini-Review. Transplant. Proc..

[7] Ghoneima A.S., Sousa Da Silva R.X., Gosteli M.A., Barlow A.D., Kron P. (2023). Outcomes of Kidney Perfusion Techniques in Transplantation from Deceased Donors: A Systematic Review and Meta-Analysis. J. Clin. Med..

[8] Hasjim B.J., Sanders J.M., Alexander M., Redfield R.R., Ichii H. (2024). Perfusion Techniques in Kidney Allograft Preservation to Reduce Ischemic Reperfusion Injury: A Systematic Review and Meta-Analysis. Antioxidants.

[9] Doménech López P., Muñoz Bastidas C., Colombás Vives J., Gutiérrez Castañé C., Chiva San Román S., Ancizu Marckert F.J., Robles García J.E. (2024). Impact of pulsatile machine perfusion on posttransplant recovery in asystole donation: Organ optimization and the future of renal transplantation. Exp. Clin. Transplant..

[10] Mahgoub M., Alrifai A.Z., Tapia A.L., Smith B., Issa N., Amer H., Dean P., Riad S. (2024). Impact of hypothermic machine perfusion on delayed graft function in kidney pairs from the same deceased donor in the United States. Transplantation.

[11] Guy A., McGrogan D., Inston N., Ready A. (2015). Hypothermic machine perfusion permits extended cold ischemia times with improved early graft function. Exp. Clin. Transplant..

[12] Kox J., Moers C., Monbaliu D., Strelniece A., Treckmann J., Jochmans I., Leuvenink H., Van Heurn E., Pirenne J., Paul A. (2018). The Benefits of Hypothermic Machine Preservation and Short Cold Ischemia Times in Deceased Donor Kidneys. Transplantation.

[13] Axelsson M., Lindnér P., Pehrsson N.G., Baid-Agrawal S. (2023). Long- and short-term effects of hypothermic machine perfusion versus cold storage on transplanted kidneys from expanded criteria donors—A matched comparison study. J. Clin. Med..

[14] Sedigh A., Tufveson G., Bäckman L., Biglarnia A.R., Lorant T. (2013). Initial experience with hypothermic machine perfusion of kidneys from deceased donors in the Uppsala region in Sweden. Transplant. Proc..

[15] Treckmann J., Moers C., Smits J.M., Gallinat A., Maathuis M.H., van Kasterop-Kutz M., Jochmans I., Homan van der Heide J.J., Squifflet J.P., van Heurn E. (2011). Machine perfusion versus cold storage for preservation of kidneys from expanded criteria donors after brain death. Transpl. Int..

[16] Gill J., Dong J., Eng M., Landsberg D., Gill J.S. (2014). Pulsatile perfusion reduces the risk of delayed graft function in deceased donor kidney transplants, irrespective of donor type and cold ischemic time. Transplantation.

[17] Verdiales C., Baxter L., Lim H.J., Beck G., Moser M.A. (2024). Matched pair analysis of the effect of longer hypothermic machine perfusion time on kidney transplant outcomes. World J. Transplant..

